# The association between the *MCM6* LCT-13910C>T variant and bone mineral density in patients with inflammatory bowel disease

**DOI:** 10.3389/fgene.2026.1822823

**Published:** 2026-07-24

**Authors:** Agata Picher, Alicja Ewa Ratajczak-Pawłowska, Marzena Skrzypczak-Zielińska, Agnieszka Dobrowolska, Iwona Krela-Kaźmierczak

**Affiliations:** 1 Institute of Human Genetics, Polish Academy of Sciences, Poznan, Poland; 2 Laboratory of Nutrigenetics, Department of Gastroenterology, Dietetics and Internal Diseases, Poznan University of Medical Sciences, Poznan, Poland; 3 Department of Gastroenterology, Dietetics and Internal Diseases, Poznan University of Medical Sciences, Poznan, Poland

**Keywords:** bone mineral density, inflammatory bowel disease, lactose intolerance, LCT-13910C>T, *MCM6* gene

## Abstract

**Background:**

Inflammatory bowel diseases (IBD), including Crohn’s disease (CD) and ulcerative colitis (UC), are associated with increased risk of osteoporosis. Beyond established environmental factors, genetic determinants and the impact of lactose intolerance on calcium–phosphate balance and bone mineralization are increasingly considered. The aim of this study was to evaluate the association between the LCT-13910C>T (rs4988235) polymorphism in the *MCM6* gene and bone mineral density (BMD) in IBD.

**Methods:**

A total of 125 patients (50 with UC, 75 with CD) and 39 healthy controls were enrolled. Genotyping was performed by high-resolution melting (HRM) analysis with Sanger sequencing confirmation for selected samples. BMD was measured at L1–L4 and at the femoral neck; selected biochemical parameters were also assessed.

**Results:**

Patients with IBD exhibited lower BMD compared with controls. Analysis of rs4988235 distribution revealed no statistically significant differences between patients and controls, although a higher frequency of the C allele and CC genotype was observed among patients. Genotype–phenotype analyses showed no significant associations for most parameters; the sole exception was the femoral neck in the CD subgroup, where CT heterozygotes had higher BMD than CC homozygotes.

**Conclusion:**

The LCT-13910C>T polymorphism does not appear to be independently associated with BMD in patients with IBD in this cohort. Given the moderate sample size and lack of adjustment for key confounders, the findings should be considered exploratory and require confirmation in larger, well-controlled studies.

## 1 Introduction

Osteoporosis is a bone disorder characterised by an increased risk of fractures due to decreased bone strength. There are two main types of osteoporosis: primary and secondary. Primary osteoporosis develops as a consequence of ageing and hormonal deficiency. On the other hand, secondary osteoporosis is caused by chronic disease or by pharmacotherapy that affects bone metabolism. One disease that may affect the bones is inflammatory bowel disease (IBD) (Szczeklik and Gajewski, 2017). Bone mineral density (BMD) is shaped by a complex interplay of non-modifiable and modifiable factors. Non-modifiable determinants include genetic background, age, and ethnicity, which collectively establish baseline skeletal integrity. In contrast, modifiable factors comprise chronic inflammation, prolonged corticosteroid exposure, low body mass index (BMI), vitamin D deficiency, inadequate calcium intake, physical inactivity, smoking, and excessive alcohol or caffeine consumption, all of which may accelerate bone loss. Many of these modifiable determinants are particularly relevant in patients with inflammatory bowel disease (IBD), in whom persistent systemic inflammation, malabsorption, and long-term steroid therapy may further compromise bone metabolism.

Patients with IBD are a group at higher risk of osteoporosis development. Decreased BMD affects 20%–50% of IBD patients (Harbord et al., 2016). Some studies reported that decreased BMD was more common in Crohn’s disease (CD) patients than in ulcerative colitis (UC) patients (Krela-Kaźmierczak et al., 2018; Tsai et al., 2015). On the other hand, the fracture risk is increased by about 40% in both patient groups (Bernstein et al., 2000; Ribaldone et al., 2020; Marzban Abbas Abadi et al., 2025). Data about the association between sex and BMD in IBD are unclear—some studies indicate a higher risk in women (Leslie et al., 2009), others in men (Jahnsen et al., 1997; Lewandowski et al., 2023), and others do not show differences between groups (Marzban Abbas Abadi et al., 2025).

**FIGURE 1:**
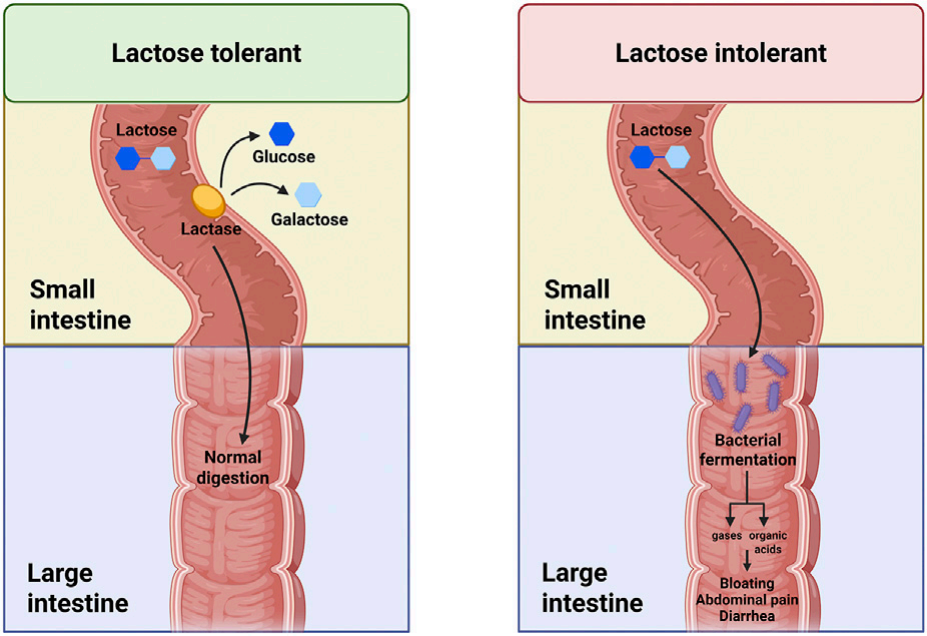
Scheme of normal and abnormal lactose digestion. Created in BioRender. Skrzypczak-zielinska, M. (2026) https://BioRender.com/94dhq9m.

Lactose is the main source of carbohydrates in cow and human milk. It is a disaccharide digested into glucose and galactose by lactase (β-D-galactosidase) in the small bowel (Flatz, 1987; Harrington and Mayberry, 2008). Lactose intolerance is one of the most common digestive of carbohydrate disorders that involves incomplete lactose breakdown caused by lactase deficiency. Non-digested lactose proceeds to the large bowel, where it is fermented by the gut microbiota. It causes the formation of organic acids and gases, causing clinical symptoms such as bloating, diarrhoea and abdominal pain (Figure 1). Intense symptoms depend on the amount of lactose intake, the severity of the deficiency and the individual patient’s tolerance (Flatz, 1987).

**FIGURE 2:**
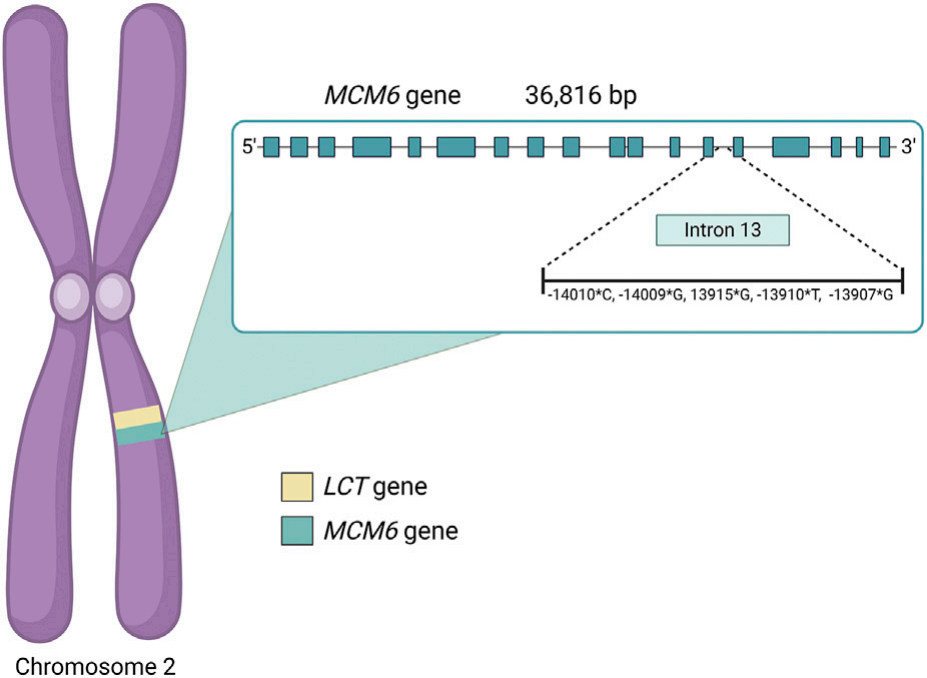
Location of the *LCT* and *MCM6* genes and key single nucleotide polymorphisms in the regulatory region of the *MCM6* gene determining lactose tolerance. Created in BioRender. Skrzypczak-zielinska, M. (2026) https://BioRender.com/j8kikb4.

Lactase activity is highest in the neonatal period and then decreases as we age. However, some people may retain the activity of this enzyme into adulthood, a condition called lactase persistence (LP). Lactase persistence is highly diversified geographically and ethnically (Goosenberg and Afzal, 2025). Primary lactose intolerance is also called adult-type hypolactasia (Heyman, 2006) and is not associated with a mutation in the gene that codes for the enzyme but rather with regulation of its expression. The *LCT* gene (locus 2q21.3) encodes lactase (Boll et al., 1991). Expression of this gene is naturally high among newborns and decreases in most mammals, including humans. *LCT* gene activity decreases significantly after milk intake ceases in most populations (Goosenberg and Afzal, 2025). *MCM6* gene, located in the immediate neighbourhood of the *LCT* gene, is the key element of regulating this process. Single-nucleotide polymorphism in the *MCM6* gene determines persistence or disappearance of lactase expression in adulthood (Järvelä, 2005) (Figure 2).

The best characterised variant is rs4988235 (−13910 C>T). The C allele is linked to primary, physiological lactose intolerance, characterised by the post-infancy loss of *LCT* gene expression. On the other hand, T allele correlates with lactase persistence and high enzyme activity in adulthood (Table 1) (Baffour-Awuah et al., 2015). The other polymorphism in the *MCM6* gene (np. rs145946881 or rs41380347) determines lactase persistence in other populations, such as Africa and the Near East (Priehodová et al., 2014; Friedrich et al., 2012; Tishkoff et al., 2007). In evolutionary terms, primary lactose intolerance is natural and original for humans (Bersaglieri et al., 2004), but it may affect the milk and dairy products intake.

**TABLE 1:** Association of the *MCM6* rs4988235 (−13910 C>T) genotype with lactose tolerance phenotype.

Genotype	Fenotype	Explanation
CC	Primary lactose intolerance	Genotype associated with a physiologically gradual decline in lactase activity after infancy
CT	Lactase persistence	One copy of the T allele is enough to keep the lactase activity in adulthood
TT	Lactase persistence	Genotype indicates the highest *LCT* gene expression and lactose tolerance

The aim of the study is to assess the association between a variant in the regulatory region of the *MCM6* gene (polymorphism LCT-13910C>T, which modulates lactase expression) and BMD in patients with IBD. Additionally, we looked for the association between genotype and certain clinical and laboratory parameters, which may influence on better understanding of the role of genetic factors in bone complications pathogenesis in patients with CD and UC.

## 2 Materials and methods

**FIGURE 3:**
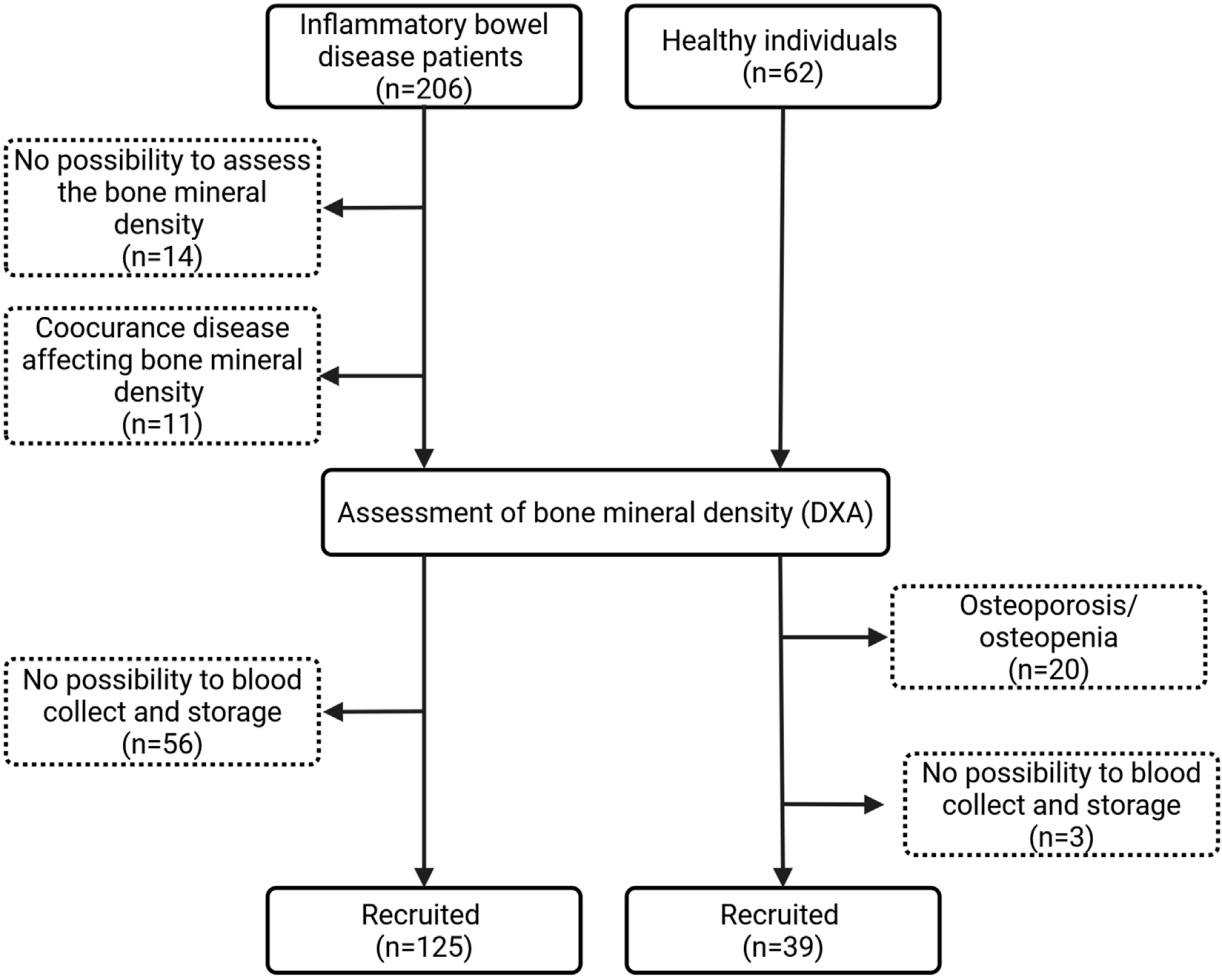
Flow chart of participant recruitment and selection, including reasons for exclusion at each stage of the study.

We enrolled 125 patients with IBD (including 75 patients with CD and 50 with UC) and 39 healthy adults (general population adults aged 18–50 years old, without any disease and drugs treatment, which may affect BMD) as a control group. A participant flow chart is shown in Figure 3. All patients were treated at the Units of Gastroenterology, Dietetics and Internal Disease or in the Gastroenterology Clinic of the Poznan University of Medical Sciences. The project was approved by the local Bioethics Committee of the Poznan University of Medical Sciences. Resolution no. 39/20 of 16th January 20.

The inclusion criteria covered:

- age between 18 and 50 years old
- diagnosed IBD based on endoscopy and histological testing
- lack of other diseases affecting BMD, especially rheumatoid arthritis, chronic kidney disease, liver disease, celiac disease or hormonal disorders
- written consent to participate in the study

All study participants were examined by Dual-energy X-ray Absorptiometry (Lunar, Inc., Madison, Wisconsin, United States) to assess the BMD, T-score and Z-score of the lumbar spine (L1–L4) and femoral neck. During the examination, we collected additional data, such as body mass and height, and calculated BMI (Body Mass Index). Moreover, we determine the concentration of calcium ionised, albumin, 25(OH)D and C-reactive protein (CRP). CD and UC patients were also assessed according to the Montreal Classification:

- For patients with CD:
  - Age at diagnosis (A)
  - Disease location (L)
  - Disease behavior (B)
- For patients with UC:
  - Extent (E)
  - Severity (S)

We also collected blood samples in EDTA tubes, from which we have isolated genomic DNA of leucocytes for genetic analysis.

### 2.1 DNA extraction and *MCM6* variants analysis

Genomic DNA was isolated from leucocytes by methods using guanidine thiocyanate (GTC) and phenol–chloroform extraction. Received DNA has been dissolved in the TE buffer.

To amplify the fragment of intron 13 of the *MCM6* gene encompassing the rs4988235 variant, specific primers were designed using the Primer3Plus tool. The primer sequences are presented in Table 2. HRM analysis was performed using the Rotor-Gene Q system (Qiagen). PCR reactions were carried out in a total volume of 16 μL, containing HRM PCR Master Mix, 0.55–2.2 μL of each primer (5 pmol/μL), and 40 ng of template DNA. Amplification conditions included an initial denaturation at 95 °C for 12 min, followed by 40 cycles of 95 °C for 15 s, 63 °C for 30 s, and 72 °C for 20 s. Melting curve analysis was conducted over a temperature range of 70 °C–90 °C with 0.1 °C increments. Data were analyzed using Rotor-Gene Q Series Software. Samples with ambiguous HRM profiles were verified by Sanger sequencing.

**TABLE 2:** Nucleotide sequences of primers used for HRM analysis and Sanger sequencing.

Gene	*MCM6* gene region	Sequences of primers (5′→3′)	Melting temperature (°C)	Chromosomal coordinates	Amplicon length (bp)
*MCM6*	Intron 13	GCCTCTGCGCTGGCAATA	63.6	chr2:135850974–135851111	138
		CCC​ACT​GAC​CTA​TCC​TCG​TG	60.5		

PCR products were enzymatically purified using the ExoSAP method with exonuclease I and shrimp alkaline phosphatase and subsequently subjected to a sequencing reaction using the BigDye® Terminator v3.1 Cycle Sequencing Kit (Thermo Fisher Scientific), according to the manufacturer’s instructions. After purification of the sequencing reaction products by ammonium acetate and ethanol precipitation, the samples were thermally denatured and sequenced in both directions using the Sanger method on an Applied Biosystems 3500 Genetic Analyzer (Applied Biosystems). The obtained chromatograms were analyzed using Chromas 2.6.6 software.

### 2.2 Statistical analysis

Normality of variable distributions was assessed using the Shapiro–Wilk test. Depending on the assumptions of normality and homogeneity of variances, intergroup comparisons were performed using one-way analysis of variance (ANOVA; with Welch’s correction in the case of unequal variances) or the nonparametric Kruskal–Wallis test. When significant differences between groups were detected, an appropriate *post hoc* test (Tukey, Games–Howell, or Dunn) was used. Associations between qualitative variables, including genotype and allele frequencies of the LCT-13910C>T variant in the *MCM6* gene, were analyzed using the chi-square test. Agreement of genotype distributions with Hardy–Weinberg equilibrium was evaluated using the Court Lab calculator (Court Lab). The final study group was selected from an initially larger cohort after applying predefined inclusion and exclusion criteria and requiring complete clinical, densitometric (DXA), and genetic data. No *a priori* sample size calculation was performed due to the exploratory nature of the study. A *post hoc* power analysis for the chi-square test of genotype distributions (Cohen’s w = 0.3, α = 0.05) indicated that the final sample size (n = 164) provided a statistical power of approximately 0.83 to detect moderate effect sizes.

All analyses were performed using GraphPad Prism v8.0.1 (GraphPad Software) and PQstat Software v.1.8.4. A p-value < 0.05 was considered statistically significant.

## 3 Results

We did not find significant differences in sex distribution among groups. The characteristics of the study group are presented in Table 3.

**TABLE 3:** Characteristics of the study groups.

Parameter	UC group (n = 50)	CD group (n = 75)	Control group (n = 39)	*p*-value		
				UC vs. control	CD vs. control	UC vs. CD
Gender, (F/M), n (%)	20/30 (40/60)	39/36 (53/48)	22/17 (56.4/43.6)	0.140	0.696	0.205
Age (year)	32.99 ± 10.21	31.78 ± 9.38	36.6 ± 7.76	0.120	<0.05	>0.999
BMI (kg/m^2^)	21.58 ± 3.81	22.13 ± 4.34	23.75 ± 3.82	<0.05	0.059	>0.999
BMD [L1-L4](g/cm^2^)	1.13 ± 0.21	1.13 ± 0.13	1.26 ± 0.11	<0.001	<0.0001	>0.999
T-score [L1–L4]	−0.53 ± 1.45	0.55 ± 1.15	0.53 ± 0.98	<0.001	<0.0001	>0.999
Z-score [L1–L4]	−0.29 ± 1.39	−0.36 ± 1.08	0.51 ± 0.94	<0.01	<0.001	>0.999
BMD [femoral neck] (g/cm^2^)	1.04 ± 0.22	1.02 ± 0.15	1.11 ± 0.08	<0.05	<0.01	>0.999
T-score [femoral neck]	−0.16 ± 1.60	−0.21 ± 1.13	0.46 ± 0.62	<0.05	<0.01	>0.999
Z-score [femoral neck]	0.17 ± 1.54	0.08 ± 1.13	0.72 ± 0.69	<0.05	<0.01	>0.999
25(OH)D level (ng/mL)	26.47 ± 13.51	29.19 ± 13.71	28.97 ± 11.33	0.683	0.997	0.640
Ionised calcium level (mg/dL)	5.14 ± 0.23	5.43 ± 1.11	5.31 ± 0.18	<0.05	0.085	>0.999
Albumin level (g/dL)	3.89 ± 0.72	4.02 ± 0.60	4.78 ± 0.26	<0.0001	<0.0001	>0.999
CRP level (mg/L)	20.39 ± 32.21	28.07 ± 43.05	1.19 ± 1.28	<0.0001	<0.0001	0.719

UC, ulcerative colitis; CD, Crohn’s disease; BMI, body mass index; BMD, bone mineral density; 25(OH)D, 25-hydroxyvitamin D; CRP, c-reactive protein.

UC and CD patients have significantly lower BMD, T-scores, and Z-scores at the lumbar spine and femoral neck than healthy controls. Patients with UC had lower ionised calcium levels than the control group. Additionally, both groups–CD and UC–have lower albumin concentrations and higher CRP levels than healthy adults.

### 3.1 Genotype and allele distribution of the LCT-13910C>T variant in patients with inflammatory bowel disease and the control group

Analysis of the rs4988235 (LCT-13910C>T) variant in the *MCM6* gene did not reveal statistically significant differences in genotype or allele distribution between patients with inflammatory bowel disease and the control group (Table 4).

**TABLE 4:** Allele and genotype distribution of rs4988235 in patients with inflammatory bowel disease and the control group.

LCT-13910C>T					
Study group	Genotype frequency (%)			Allele frequency (%)	
	TT	CT	CC	T	C
IBD patients (n = 125)	26 (20.8%)	58 (46.4%)	41 (32.8%)	110 (44%)	140 (56%)
Control group (n = 39)	11 (28.2%)	20 (51.3%)	8 (20.5%)	42 (53.9%)	36 (46.2%)
IBD vs. Control group	[TT] vs. [CC] OR 2.2 95% CI 0.81–6.41 p = 0.138	[TT + CT] vs. [CC] OR 0.5 95% CI 0.23–1.23 p = 0.143	[CT + CC] vs. [TT] OR 1.5 95% CI 0.69–3.42 p = 0.334	T allele vs. C allele OR 1.5 95% CI 0.89–2.50 p = 0.128	

The frequency of the C allele was higher in the IBD group compared to controls (56% vs. 46.2%), while the T allele was less frequent (44% vs. 53.9%). Similarly, the CC genotype was more common among IBD patients (32.8% vs. 20.5%), whereas the TT genotype occurred less frequently (20.8% vs. 28.2%). However, these differences did not reach statistical significance.

Therefore, no significant association between the rs4988235 variant and IBD status was observed in the studied population.

Genotype distributions in both groups were consistent with Hardy–Weinberg equilibrium (control group: p = 0.723; IBD group: p = 0.514), indicating no deviation from expected genetic proportions.

### 3.2 Genotype and allele distribution of the LCT-13910C>T variant in patients with ulcerative colitis and the control group

The distribution of the rs4988235 (LCT-13910C>T) variant in the *MCM6* gene did not show statistically significant differences in genotype or allele frequencies between patients with ulcerative colitis and the control group (Table 5).

**TABLE 5:** Allele and genotype distribution of rs4988235 in patients with ulcerative colitis and the control group.

LCT-13910C>T					
Study group	Genotype frequency (%)			Allele frequency (%)	
	TT	CT	CC	T	C
UC patients (n = 125)	12 (24%)	20 (40%)	18 (36%)	44 (44%)	56 (56%)
Control group (n = 39)	11 (28.2%)	20 (51.3%)	8 (20.5%)	42 (53.9%)	36 (46.2%)
UC vs. Control group	[TT] vs. [CC] OR 2.1 95% CI 0.61–6.33 p = 0.221	[TT + CT] vs. [CC] OR 0.5 95% CI 0.17–1.15 p = 0.111	[CT + CC] vs. [TT] OR 1.2 95% CI 0.46–3.26 p = 0.653	T allele vs. C allele OR 1.5 95% CI 0.82–2.73 p = 0.192	

UC, ulcerative colitis; OR, odds ratio; 95% CI, confidence interval.

In the UC group, the C allele was present in 56% of cases, compared to 46.2% in controls, while the T allele was less frequent (44% vs. 53.9%, respectively). The proportion of individuals with the CC genotype was higher among UC patients (36%) than in the control group (20.5%), whereas the TT genotype was observed less frequently (24% vs. 28.2%).

Therefore, no significant association was observed between the rs4988235 variant and the occurrence of UC in the study population.

The genotype distributions were consistent with the Hardy–Weinberg equilibrium in both groups (control group: p = 0.723; UC group: p = 0.183), indicating that the observed frequencies did not deviate from expected proportions.

### 3.3 Genotype and allele distribution of the LCT-13910C>T variant in patients with Crohn’s disease and the control group

The distribution of the rs4988235 (LCT-13910C>T) variant in the *MCM6* gene in patients with Crohn’s disease was comparable to that observed in the overall IBD and UC groups. No statistically significant differences in genotype or allele frequencies were identified between CD patients and controls (Table 6).

**TABLE 6:** Allele and genotype distribution of rs4988235 in patients with Crohn’s disease and the control group.

LCT-13910C>T					
Study group	Genotype frequency (%)			Allele frequency (%)	
	TT	CT	CC	T	C
CD patients (n = 75)	14 (18.7%)	38 (50.7%)	23 (30.7%)	66 (44%)	84 (56%)
Control group (n = 39)	11 (28.2%)	20 (51.3%)	8 (20.5%)	42 (53.9%)	36 (46.2%)
CD vs. Control group	[TT] vs. [CC] OR 2.3 95% CI 0.73–6.42 p = 0.153	[TT + CT] vs. [CC] OR 0.64 95% CI 0.24–1.43 p = 0.248	[CT + CC] vs. [TT] OR 1.7 95% CI 0.69–4.02 p = 0.243	T allele vs. C allele OR 1.5 95% CI 0.86–2.60 p = 0.158	

CD, Crohn’s disease; OR, odds ratio; 95% CI, confidence interval.

The C allele was more frequent in the CD group than in controls (56% vs. 46.2%), while the CC genotype was also observed more often (30.7% vs. 20.5%). However, these differences did not reach statistical significance.

Genotype distributions in both groups were consistent with Hardy–Weinberg equilibrium (control group: p = 0.723; CD group: p = 0.807), indicating no deviation from expected genetic proportions.

### 3.4 Analysis of the relationship between genotypes and clinical and laboratory parameters

In the IBD and UC patient group, we did not find any statistically significant association between the analysed genotypes and clinical or laboratory parameters, including BMI, ionised calcium, 25(OH)D, albumin, CRP, BMD, T-score, and Z-score of the femoral neck and L1–L4.

Similar results were obtained in the CD group, except for femoral neck BMD. A statistically significant difference was observed between CT heterozygotes and CC homozygotes (p = 0.043), with higher BMD values in CT individuals. This finding should be interpreted with caution, as it was not supported by other comparisons and may represent a chance observation. No statistically significant differences were found between TT and CC homozygotes (p = 0.217) or between CT and TT genotypes (p = 0.950) (Table 7).

**TABLE 7:** Bone mineral density of the femoral neck depends on rs4988235 variant in patients with Crohn’s disease.

Genotype	n	BMD of the femoral neck [mean ± SD] (g/cm^2^)	*P* - value
Heterozygotes CT	37	1.057 ± 0.1692	0.043^a^ 0.950^b^ 0.217^c^
Homozygotes TT	13	1.042 ± 0.1365	
Homozygotes CC	23	0.964 ± 0.1213	

^a^ CT vs. CC.

^b^ CT vs. TT.

^c^ TT vs. CC.

## 4 Discussion

Osteoporosis is a significant clinical problem in patients with inflammatory bowel disease, as it is associated with an increased risk of fractures, which may lead to complications such as immobilisation, muscle atrophy, pressure ulcers, pulmonary embolism, increased susceptibility to infections, and increased mortality in older individuals (Krela-Kaźmierczak et al., 2016). Reduced bone mineral density is observed in approximately 20%–50% of IBD patients, with a 40%–60% higher risk of fractures compared to the healthy population (Harbord et al., 2016; Argollo et al., 2019).

Risk factors of osteoporosis in IBD include malnutrition related to the disease and corticosteroid therapy. Increasing evidence also suggests that genetic factors may contribute to bone metabolism disorders (Adriani et al., 2014). In the present study, no significant differences in 25(OH)D or ionised calcium levels were observed between IBD patients and controls, except for lower ionised calcium levels in the UC group (p < 0.05). At the same time, patients with IBD demonstrated lower BMD, T-scores, and Z-scores at both the femoral neck and L1–L4, indicating impaired bone mineralisation. These findings suggest that, in this cohort, bone alterations were not solely explained by deficiencies in vitamin D or calcium, supporting the potential involvement of additional factors, including genetic determinants.

Both osteoporosis and IBD are polygenic diseases, and inflammation is an important factor in their pathogenesis. LCT-13910C>T variant in *the MCM6* gene is a well-established genetic determinant of lactase persistence in populations of European origin, influencing the ability to digest lactose. Individuals with lactose intolerance often eliminate dairy products from their diet, which may reduce calcium and vitamin D intake and subsequently affect bone metabolism. For this reason, this polymorphism was selected to evaluate its association with BMD in patients with IBD.

Analysis of the rs4988235 variant in the *MCM6* gene did not show significant differences in genotype or allele frequencies between IBD patients and the control group. However, a higher frequency of the C allele and the CC genotype was observed in patients with IBD, UC, and CD compared with controls. These differences did not reach statistical significance and should therefore be interpreted with caution, as they may indicate only a possible trend rather than a true association with disease susceptibility. Notwithstanding, these findings are in line with the study by Elguezabal et al., which also did not demonstrate a clear association between the LCT-13910C>T genotype and IBD, although a higher frequency of the C allele in UC patients was reported (Elguezabal et al., 2012).

A similar trend was reported by Mnich et al. (Mnich et al., 2018). They analysed medieval bone samples from Polish territory and observed a lower BMD among CC heterozygotes. This study also showed that people with lactase deficiency are more likely to have osteoporosis, but neither observation was statistically significant. Another study among the Polish population also found that nutritional status was similar between people with lactose intolerance and the control group, whereas bone mass was lower in the group with the CC genotype, but these differences were also statistically insignificant (Kowalówka et al., 2023). Taken together, the available evidence suggests a possible influence of the CC genotype on bone metabolism; however, current findings remain inconclusive and require confirmation in larger cohorts.

The results of this study should be interpreted in the context of geographical and ethnic differences in the frequency of the LCT-13910CC genotype. For instance, the frequency observed in Chilean IBD patients (61%) corresponds to that reported for the general North and South American population (63.1%) (Pérez-Jeldres et al., 2023), suggesting that it reflects population genetics rather than disease association. In the present study, the frequency of the CC genotype in Polish IBD patients (32.8%) was comparable to that reported for European (∼30.6%) and Polish populations (∼31.5%) (Mądry et al., 2010).

It should also be noted that genetic testing identifies a predisposition (the presence of the CC genotype) to primary lactose intolerance, but does not assess lactase activity directly. Therefore, a genetic test result does not necessarily indicate the ability or disability to digest lactose. In clinical practice, concordance between the CC genotype and lactose intolerance confirmation by breath hydrogen test is high, as many studies have shown. In one study, 100% of patients with lactose intolerance confirmed by breath hydrogen test were carriers of the 13910CC genotype. At the same time, genotype 13910CC was observed in only 17% of patients with normal lactose tolerance, emphasising the high predictive value of this gene variant for hypolactase (Bernardes-Silva et al., 2007). In another study, the 13910CC genotype occurred in 88% of patients with abnormal breath hydrogen test results and in none with normal tolerance (Krawczyk et al., 2008). Breath hydrogen test assesses the current ability to digest lactose: positive results with the CC genotype indicate primary lactose intolerance; positive results with the CT or TT genotypes suggest secondary lactose intolerance, for example, associated with small intestinal mucosal damage. However, a CT heterozygote may have an intermediate lactase activity—higher than a person with the CC genotype, and lower than a TT homozygote (Enattah et al., 2007). Additionally, results of the breath hydrogen test may be falsified by other gastrointestinal tract disorders (Eadala et al., 2011; Misselwitz et al., 2019).

Lactose intolerance (both primary and secondary) is also an important aspect of clinical care among IBD patients, which often involves eliminating milk and dairy products to avoid gastrointestinal symptoms (Triggs et al., 2010; Di Stefano et al., 2002). It leads to a decrease in the intake of calcium, potassium, vitamin D, vitamins B and protein (Górska-Warsewicz et al., 2019). In Poland, dairy products are the main source of calcium in the diet, accounting for about 50% of daily calcium intake (Rychlik et al., 2024; Rozenberg et al., 2015). Elimination of them may increase the risk of osteopenia and osteoporosis development in IBD patients (Di Stefano et al., 2002; Falbová et al., 2025).

The *MCM6* rs4988235 (LCT-13910C>T) variant is a well-established genetic determinant of lactase persistence and may influence lactose digestion capacity. Through its effect on dairy product consumption, this variant may indirectly influence calcium intake and, consequently, bone mineralization. However, the present study did not include direct assessment of lactose intolerance or detailed dietary data, and therefore the genotype–phenotype–BMD relationship could not be fully elucidated. Bone metabolism in IBD is multifactorial and influenced by nutritional, inflammatory, and treatment-related factors, which may further modify this relationship. Although no direct association with BMD was demonstrated in this study, an indirect effect mediated by dietary habits cannot be excluded. This highlights the importance of considering gene–environment interactions when interpreting genetic association studies.

This concept is supported by a large cohort study by Falbová et al. (2025) (n = 785) which demonstrated that both self-reported lactose intolerance and lifestyle-related factors significantly influence bone mineral density in young adults. Importantly, environmental and lifestyle variables may considerably confound the assessment of the relationship between lactose intolerance and BMD, suggesting that behavioral phenotypes, such as dietary restrictions, may be more relevant determinants of bone health than genetic variants alone. These findings underscore the importance of multivariable approaches to disentangle the effects of genetic and non-genetic factors (Falbová et al., 2025).

To reduce the risk of malnutrition, IBD patients with lactose intolerance should eat dairy products with reduced lactose content (e.g., fermented dairy products such as kefir, yoghurt, and cheese) or use a lactase supplement. A good source of calcium may also be plant milk (e.g., rice, soy, or coconut), which is fortified with calcium and vitamin D. Additionally, a source of calcium may be green leafy vegetables (Ratajczak et al., 2021).

Although we paid attention to proper methodology, we are aware of some limitations of this study. Firstly, detailed data on dietary calcium and vitamin D intake were not available, and information on dairy product consumption was not collected, limiting the ability to assess diet–genotype interactions.

Both dietary calcium and vitamin D are important factors of bone mineral density and the intake of these nutrients affects bone metabolism significantly (Malpeli et al., 2012; Moschonis and Manios, 2006). However, lactose intolerance may also influence dairy products consumption and affect total calcium and vitamin D intake (Heaney, 2013). Second, data on pharmacotherapy, particularly corticosteroid exposure, were not comprehensively assessed despite their known impact on bone metabolism. The study presented that IBD patients with lifetime dose of corticosteroids (prednisone/prednisolone) higher than 10 g had lower BMD that patients with low steroids dose (below 5 g) (Silvennoinen et al., 1995) Therefore, steroid use is one of the most important factors affecting bone in IBD. However, collecting the data about lifetime steroid use is really hard, and sometimes impossible (due to lack of medical documentation).

Third, the analysis was limited to a single genetic variant, while bone metabolism is influenced by multiple genes (e.g., *VDR, COL1A1*). In addition, information on lifestyle factors, such as physical activity and sunlight exposure, was not available. However, it is well-known that physical activity influences bone positively (Sanchez-Trigo et al., 2025). In fact, assessment of physical activity in an observational study is often impossible. Many validated questionnaires focus only on a specific period and do not account for physical activity throughout life.

Finally, although *post hoc* power analysis indicated that the study was adequately powered to detect moderate genetic effects, the sample size was moderate and may have been insufficient to detect smaller associations. Moreover, formal correction for multiple testing was not applied due to the exploratory nature of the study, which may increase the risk of type I error. This is particularly relevant for the single statistically significant association observed in the CD subgroup, which may represent a chance finding. Despite the above limitation, this study provides important information about the possible role of LCT-13910C>T polymorphism in bone metabolism among patients suffering from IBD and is the starting point for future analysis with broader clinical, environmental and genetic context.

## 5 Conclusion

In conclusion, the LCT-13910C>T polymorphism does not appear to be directly associated with bone metabolism in patients with IBD. However, it may indirectly affect bone health through its impact on lactose intolerance and related dietary habits. Identification of patients with lactose intolerance using clinical assessment, genetic testing, and hydrogen breath testing may help identify individuals who require closer monitoring and preventive strategies for osteoporosis. These patients may benefit from targeted dietary counseling and education regarding appropriate calcium and vitamin D intake. Monitoring of BMD, along with proper dietary management and supplementation, may reduce the risk of bone complications in this population.

## Data Availability

The data presented in the study are publicly available in the zenodo repository, with the accession number https://doi.org/10.5281/zenodo.21261597.
